# Metadata-guided feature disentanglement for functional genomics

**DOI:** 10.1093/bioinformatics/btae403

**Published:** 2024-09-04

**Authors:** Alexander Rakowski, Remo Monti, Viktoriia Huryn, Marta Lemanczyk, Uwe Ohler, Christoph Lippert

**Affiliations:** Digital Health Machine Learning, Hasso Plattner Institute for Digital Engineering, Digital Engineering, University of Potsdam, Campus III Building G2, Rudolf-Breitscheid-Strasse 187, Potsdam, Brandenburg, 14482, Germany; Digital Health Machine Learning, Hasso Plattner Institute for Digital Engineering, Digital Engineering, University of Potsdam, Campus III Building G2, Rudolf-Breitscheid-Strasse 187, Potsdam, Brandenburg, 14482, Germany; Max-Delbrück-Center for Molecular Medicine in the Helmholtz Association, Berlin Institute for Medical Systems Biology, Department of Biology, Humboldt Universität Berlin, Hannoversche Strasse 28, Building 101, Room 1.05, Berlin, 10115, Germany; Max-Delbrück-Center for Molecular Medicine in the Helmholtz Association, Berlin Institute for Medical Systems Biology, Department of Biology, Humboldt Universität Berlin, Hannoversche Strasse 28, Building 101, Room 1.05, Berlin, 10115, Germany; Data Analytics and Computational Statistics, Hasso Plattner Institute for Digital Engineering, Digital Engineering, University of Potsdam, Potsdam, Brandenburg, 14482, Germany; Max-Delbrück-Center for Molecular Medicine in the Helmholtz Association, Berlin Institute for Medical Systems Biology, Department of Biology, Humboldt Universität Berlin, Hannoversche Strasse 28, Building 101, Room 1.05, Berlin, 10115, Germany; Digital Health Machine Learning, Hasso Plattner Institute for Digital Engineering, Digital Engineering, University of Potsdam, Campus III Building G2, Rudolf-Breitscheid-Strasse 187, Potsdam, Brandenburg, 14482, Germany; Hasso Plattner Institute for Digital Health at Mount Sinai, Icahn School of Medicine at Mount Sinai, New York, NY, 10029, United States of America

## Abstract

**Summary:** With the development of high-throughput technologies, genomics datasets rapidly grow in size, including functional genomics data. This has allowed the training of large Deep Learning (DL) models to predict epigenetic readouts, such as protein binding or histone modifications, from genome sequences. However, large dataset sizes come at a price of data consistency, often aggregating results from a large number of studies, conducted under varying experimental conditions. While data from large-scale consortia are useful as they allow studying the effects of different biological conditions, they can also contain unwanted biases from confounding experimental factors. Here, we introduce Metadata-guided Feature Disentanglement (MFD)—an approach that allows disentangling biologically relevant features from potential technical biases. MFD incorporates target metadata into model training, by conditioning weights of the model output layer on different experimental factors. It then separates the factors into disjoint groups and enforces independence of the corresponding feature subspaces with an adversarially learned penalty. We show that the metadata-driven disentanglement approach allows for better model introspection, by connecting latent features to experimental factors, without compromising, or even improving performance in downstream tasks, such as enhancer prediction, or genetic variant discovery. The code will be made available at https://github.com/HealthML/MFD.

## 1 Introduction

Consortia such as Encyclopedia of DNA Elements (ENCODE) (Luo *et al.* 2019) have accumulated a wealth of high-throughput functional genomics data across a broad range of cell lines, developmental time points, and tissues, for instance measuring chromatin modifications and DNA accessibility. These data have spurred the development of deep neural networks (DNNs) that predict the readouts of these experiments from DNA sequence inputs to better understand the sequence features that govern gene regulation (Zhou and Troyanskaya 2015, Kelley *et al.* 2016, Avsec *et al.* 2021b).

The development of Explainable Artificial Intelligence (XAI) methods has allowed for assessing the importance of input features for deep learning (DL) models’ predictions. A commonly used approach to interpret genomic DL models comprises post hoc interpretation methods, producing sequence attribution maps (for an overview, see Novakovsky *et al.* 2023). However, these maps have been shown to produce spurious results (Hooker *et al.* 2019). Although properties of the learned function and the particularities of the methods themselves have been identified as contributing to noisy attributions, and solutions have been proposed (Majdandzic *et al.* 2023), these do not tackle the issue of noise in the training data.

Genomics data are heavily affected by experiment-specific (e.g. selectivity of DNA restriction enzymes) and technology-specific (e.g. adapter choice, amplification method) biases as well as strong batch effects (e.g. laboratories, vendors; Leek and Storey 2007). These biases mask intended signals and affect downstream analyses. Proposed correction methods usually address only specific sets of biases and have not become widely used in practice (Wang *et al.* 2017). Recent work has demonstrated the utility of XAI to uncover biases in genomics training data (Ghanbari and Ohler 2020), which indicates that genomics models may heavily rely on biases in addition to genuine biological features to make predictions. It is unclear how strongly this affects downstream applications, such as enhancer sequence or genetic variant effect prediction (VEP). Therefore, directly modeling sources of bias and employing inherently interpretable model designs should contribute to overcoming these issues and improving downstream task performance.

Disentangled Representation Learning (DRL) focuses on separating the generative factors underlying the observable data (Bengio *et al.* 2013) by imposing properties on a learned latent data representation space, e.g. conditionally factorizable priors (Khemakhem *et al.* 2020, Locatello *et al.* 2020), or imposing invariance to a set of variables (Ganin *et al.* 2016, Zhao *et al.* 2020, Adeli *et al.* 2021, He and Xie 2021). The recently introduced method, Disentangled Relevant Subspace Analysis (DRSA) (Chormai *et al.* 2022), enhances the interpretability of machine learning models by working in conjunction with XAI techniques. DRSA focuses on analyzing relevant subspaces within a model’s activation layers rather than solely examining the final predictions. This approach separates and clarifies the contributions of various features to model decisions, enhancing transparency and understanding of complex datasets.

In the context of biomedical applications, DRL models demonstrate increased explainability, robustness, and better generalization (Schreiber *et al.* 2020, Yang *et al.* 2022, Lotfollahi *et al.* 2023). Such approaches typically require information on a per-observation level, typically in the form of additional observed variables. Instead, we consider a setting where the auxiliary information is not available per-observation, but we have access to metadata defining relations between different classes of outcomes.

To this end, we propose Metadata-guided Feature Disentanglement (MFD)—a DNN DNA sequence model that leverages metadata of the predictions of interest, in our case metadata from ENCODE experiments, to separate biological features from technical ones by learning two independent latent subspaces. We train MFD on human genome data to predict peak calls from 2106 ENCODE experimental tracks, and we demonstrate its impact on model interpretability (Section 3.1) and downstream task performance on independent data (Sections 3.2 and 3.3).

## 2 Metadata-guided feature disentanglement

MFD is a DL model predicting peak calls of multiple tissue-based experiments from DNA sequence data while learning two disentangled feature sub-spaces, corresponding to biological and technical experiment metadata. It consists of three modules: (i) a Convolutional Neural Network (CNN) sequence feature extractor, based on the Basenji2 architecture (Avsec *et al.* 2021a) (ii) a metadata embedding module based on two static hypernetworks (Ha *et al.* 2016) mapping the metadata of each experiment to a set of weights, which are in turn used to compute the corresponding peak prediction from the extracted DNA features (Section 2.1) (iii) a regularization penalty, enforcing independence between the two latent sub-spaces of the model (Section 2.2). Model training and data collection are described in Supplementary Appendix Sections A and C.

### 2.1 Metadata embeddings

**Figure 1.**
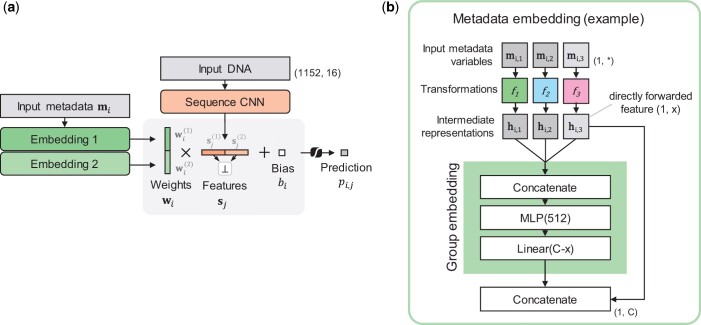
Model architecture and example of the metadata embedding module. (a) Variables in metadata row $m_{i}$ for class *i* are fed through two metadata embedding modules 1 and 2 to produce weights $w_{i}^{\left(1\right)}$ and $w_{i}^{\left(2\right)}$, with $w_{i}=[w_{i}^{\left(1\right)},w_{i}^{\left(2\right)}]$. The sequence CNN extracts sequence features $s_{j}$ from the 1152 bp sequence. Weights $w_{i}$ are multiplied with $s_{j}$, a bias *b_i_* is added, and the sigmoid activation function is applied to produce the prediction $p_{i,j}$. A penalty is placed on the Mutual Information between features in $s_{j}^{\left(1\right)}$ and $s_{j}^{\left(2\right)}$ (⊥) in order to enforce independence between the two latent subspaces. (b) A metadata embedding module with three variables $m_{i,1}$–$m_{i,3}$ (vectors or scalars), which are transformed by functions *f*_1_–*f*_3_ to produce intermediate variables $h_{i,1}$–$h_{i,3}$. The module can learn interactions between variables by feeding them through an MLP (Supplementary Fig. S5), followed by a linear mapping to *C−x* dimensions. The metadata variable 3 with intermediate dimension 1×x is directly forwarded and concatenated to yield *C* weights in total.

We integrate the experiment metadata as follows: the metadata matrix $M\in R^{O\times M}$ is non-linearly transformed via metadata embeddings—trainable Multilayer Perceptrons (MLPs)—to derive weights of the output layer of the network $W\in R^{C\times O}$, where *C* is the number of latent features from the sequence model, *M* is the number of metadata variables, and *O* is the number of experiments. To produce a single prediction $p_{i,j}$ for class *i* and sequence *j*, the corresponding row in the weights matrix $w_{i}$ is multiplied with the sequence representation $s_{j}\in R^{1\times C}$, a class-specific bias (*b_i_*) is added, and a sigmoid activation is applied (Fig. 1):

(1) $$p_{i,j}=\sigma \left(s_{j}w_{i}+b_{i}\right).$$

We divide the metadata variables into two groups, loosely interpretable as biological (e.g. tissue type, life stage, target) or technical (e.g. year, facility) experimental factors. The set of biological features is motivated by the fact that different tissues have distinct genetic programs that change during an organism’s development and, therefore, will differ in epigenetic targets (e.g. whether DNA is accessible or if a repressive mark is present). In turn, technical features contain information about biases that arise from experimental procedures and batch effects. We train a separate embedding module $\psi^{\left(i\right)}:R^{M^{\left(i\right)}}\;\mapsto \;R^{C/2},i\in \{1,2\}$ for each feature group. The two resulting sets of weights $w^{\left(1\right)}$ and $w^{\left(2\right)}$ are separately applied to the first and second halves of the extracted sequence features $s_{j}^{\left(1\right)}$ and $s_{j}^{\left(2\right)}$:

(2) $$\begin{array}{c} p_{i,j}=\sigma \left(s_{j}^{\left(1\right)}\psi^{\left(1\right)}{\left(M\right)}_{i}+s_{j}^{\left(2\right)}\psi^{\left(2\right)}{\left(M\right)}_{i}+b_{i}\right)\\ =\sigma \left(s_{j}^{\left(1\right)}w_{i}^{\left(1\right)}+s_{j}^{\left(2\right)}w_{i}^{\left(2\right)}+b_{i}\right). \end{array}$$

This means that the biological metadata variables can only influence the final predictions via features from the first subset $s^{\left(1\right)}$, while technical metadata can only utilize features from $s^{\left(2\right)}$. We further note that the metadata embeddings have an additional regularizing effect, as two classes with identical metadata are considered replicates, and share the same weights *w_i_* in the output layer—their predictions differ only by their class biases.

### 2.2 Learning independent subspaces

In order to learn disjoint features for the two latent subspaces, we additionally train the model to minimize the Mutual Information (MI) between the biological and technical feature subspaces, using an adversarial training approach. We train two MLPs models, denoted as $\varrho_{1-2}$ and $\varrho_{2-1}:R^{C/2}\;\mapsto \;R^{C/2}$, to predict biological features from the technical ones, and vice-versa. Specifically, during the adversarial training step we minimize:

(3) $$\begin{array}{c} L_{\text{indep}}=-\sum_{i}^{C/2}{\left[\rho_{\left(i\right)}\left(s^{\left(2\right)},\varrho_{1-2}(s^{\left(1\right)})\right)\right]}^{2}-\\ -\sum_{i}^{C/2}{\left[\rho_{\left(i\right)}\left(s^{\left(1\right)},\varrho_{2-1}(s^{\left(2\right)})\right)\right]}^{2}, \end{array}$$

where with $\rho_{\left(i\right)}(x,y)$ we denote the Pearson’s correlation between the *i*th dimensions of **x** and **y** computed empirically over a mini-batch of samples. Consequently, the objective for the training step of the sequence model becomes:

(4) $$\begin{array}{c} L_{\text{MFD}}=-\lambda_{\text{indep}}L_{\text{indep}}+\\ \frac{1}{NO}\sum_{j=1}^{O}\sum_{i=1}^{N}y_{i,j}\;\;\text{log}(p_{i,j})+(1-y_{i,j})\;\;\text{log}(1-p_{i,j}), \end{array}$$

where $y_{i,j}$ is the binary label of the *j*th class for the *i*th mini-batch sample, and *λ*_indep_ controls the strength of the subspace-independence penalty. Employing the independence penalty in the form of adversarially trained predictors, as opposed to, e.g. a cross-covariance penalty, ensures the independence of the subspaces in a general sense, constrained only by the capacity of *ϱ*, and not limited to simple linear dependencies (see Supplementary Appendix Section B).

## 3 Results

Here we demonstrate how MFD allows for increased interpretability, by linking latent DL features to different experimental factors (Section 3.1), while retaining or even improving performance on downstream tasks such as enhancer prediction (Section 3.2) and VEP (Section 3.3), as compared to a baseline model without metadata and independence constraints. All the results are obtained with models pretrained on the ENCODE data (Supplementary Appendix Section A).

### 3.1 MFD enables interpretation of experimental factors

To determine what the latent subspaces learned, we interpret the models by using Integrated Gradients (Sundararajan *et al.* 2017). To this end, we apply the neuron attribution implementation from the Captum package (Kokhlikyan *et al.* 2020) to each node in the latent subspace layer to determine contribution scores for each position in the input sequence. Since the sequences are dinucleotide-encoded, we assign the contribution score to the first nucleotide of the two nucleotides, which corresponds to the nucleotide at the given position.

**Figure 2.**
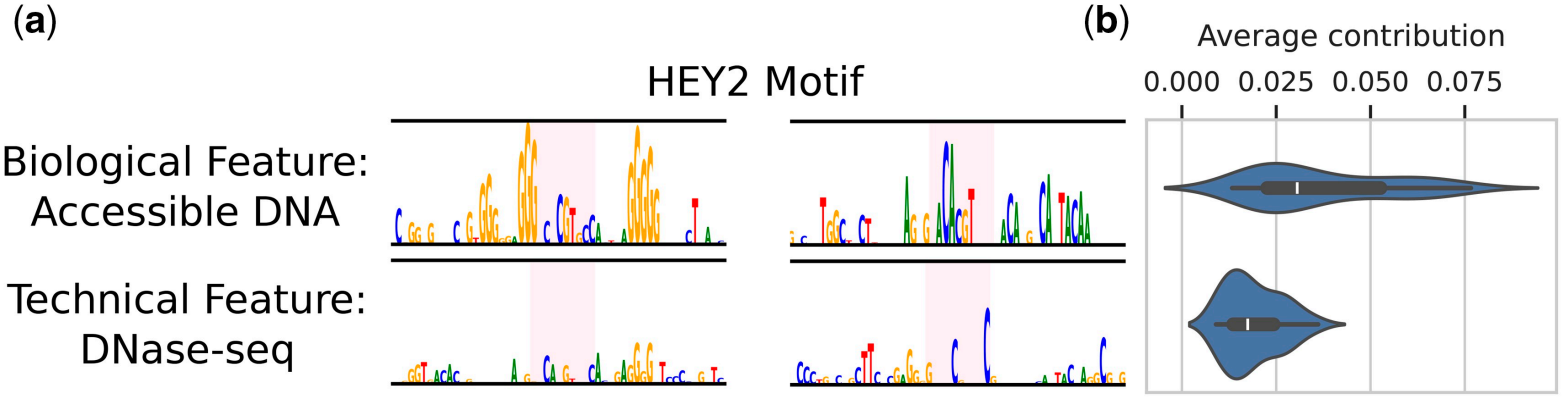
Exemplary case of interpretability: contribution scores for direct features corresponding to “Accessible DNA” (biological) and “DNase-seq” (technical). (a) The highlighted region represents the HEY2 motif for two sample sequences. (b) The average contribution scores for the context region ±50 bp around the motif for sequences with the HEY2 motif within the central 128 bp (*n* = 40). We show how MFD allows for the interpretation and comparison of how input sequences interact with different experimental factors, using features directly corresponding to metadata factors. The “DNase-seq” feature is sensitive to different characteristics around the HEY2 motif than the “Accessible DNA” feature (a), and is overall less influenced by the motif (b).

As an example case, we evaluate contribution scores for sequences with the HEY2 Transcription Factor (TF)-binding motif. HEY2 is known to be a regulator of early heart development. We select regions from test chromosomes that have HEY2 binding motifs and focus on the biological target feature, “Accessible DNA,” and the technical feature, “DNase-seq” (Fig. 2). DNase-seq is an experimental procedure to measure DNA accessibility or “openness” that is often interpreted as sequence activity. The motif is present in the attribution maps for the biological feature, while it cannot be observed in those for the technical feature for the same input sequence. The average contribution for sequences with the HEY2 motif within the central 128 bp window varies between the features. This indicates that the subspaces capture different signals and confirms that the “Accessible DNA” feature attends to biologically meaningful motifs.

**Figure 3.**
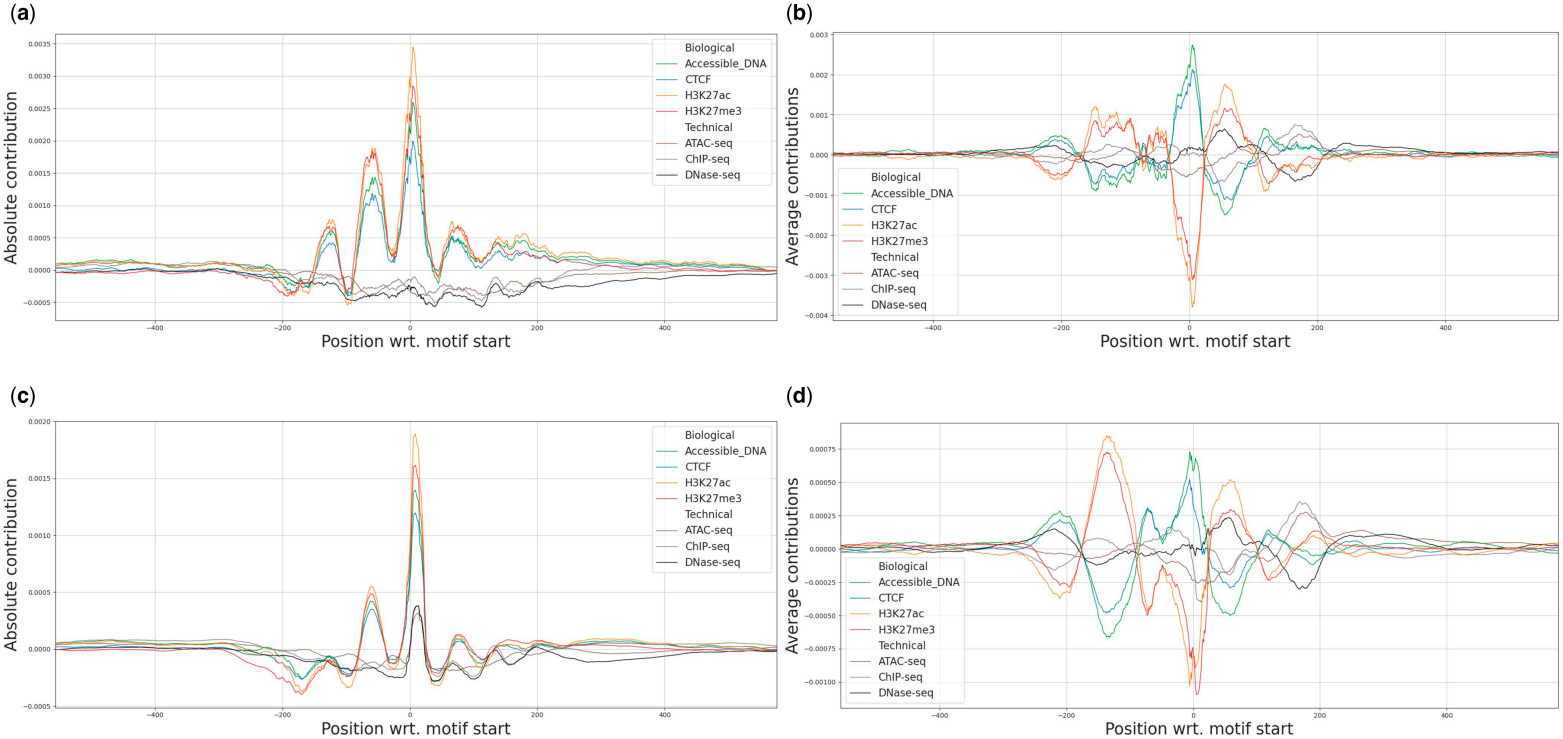
Contribution scores w.r.t. position in input sequence for direct metadata features for targets and assays (a) absolute and (b) average scores per base pair positions for 100 sequences with footprints for heart tissue (c) absolute and (d) average scores per base pair positions for 4457 sequences with CTCF motifs. We subtracted a “baseline” signal from all examples, computed from 4569 sequences which had no corresponding experiment peaks. Using features directly corresponding to metadata factors allows us to interpret model predictions on a finer scale. For example, features corresponding to assay type seem to ignore the heart TFs motifs (a, b), while they seem sensitive to the CTCF ones (c, d), as indicated by the peaks around the start of the CTCF motifs. Furthermore, the features of histone modifications (H3K27ac, H3K27me3) react in the opposite direction than features of CTCF and Acc. DNA (b, d).

Furthermore, we examine attribution scores for sequences from test chromosomes with identified TF footprints. Footprints were previously identified using DNase-seq experiments from ENCODE (Bentsen *et al.* 2020). They indicate short 16 nucleotide-long regions of estimated TF binding sites. We compute contribution scores for the directly forwarded features for targets (Accessible DNA, CTCF, H3K27ac, H3K27me3) and assays (DNase-seq, ATAC-seq, ChIP-seq) for two groups of sequences. The first group consists of 100 sequences, each centered on a unique high-score footprint with no other high-score footprints within 400 nucleotides upstream or downstream of the center (Fig. 3a and b). The second group consists of 4457 sequences centered on footprints containing a CTCF binding motif (Fig. 3c and d). CTCF is a ubiquitous TF present in all cell types. We also calculate attribution scores for “baseline” sequences, defined as those exhibiting fewer than two signal peaks across all ENCODE experiments used as classes in training, and thus having no TF footprints, and subtract them from the motif contributions, in order to separate motif-specific contributions from the baseline signal of the model (see Fig. A7 of the Supplementary Appendix for examples of the baseline signal and uncorrected plots). Resulting plots show that the center of the sequence (the footprint) has high attribution scores for biological features such as Accessible DNA and CTCF and lower for technical features. This suggests that latent biological features correspond to meaningful biological signals within the input sequences. However, the observed periodical pattern, especially visible in the absolute contribution plots, might be an artifact of convolutional layers of the model.

### 3.2 Biological features suffice for enhancer prediction

With trained DRL models at hand, we reason that the learned separation of latent subspaces into biological and technical can provide more robust features for downstream tasks. To evaluate this, we set up binary classification tasks to predict enhancer activity in the FANTOM5 dataset (Dalby *et al.* 2017) and enhancer presence in the Vista dataset (Visel *et al.* 2007a). We encode the sequences using pretrained MFD models, obtaining three sets of features: biological $s_{\text{bio}}\in R^{N\times C}$, technical $s_{\text{tech}}\in R^{N\times C}$, and combined $s_{\text{full}}=[s_{\text{bio}},s_{\text{tech}}]\in R^{N\times 2C}$. Each sequence is encoded in both the forward as well as the reverse directions, and the corresponding features are concatenated, resulting in *C* features per subspace (instead of C/2). Features obtained this way serve as inputs for regularized logistic regression models to predict the probability of a DNA sequence being an enhancer. For each tissue type, we train and evaluate 12 Ridge logistic regression models using MFD features: three feature types ($s_{\text{bio}},s_{\text{tech}},s_{\text{full}}$) × four MFD models trained with different values of *λ*_indep_ (see Supplementary Appendix E for more details). Additionally, we evaluate features from a baseline model without metadata embeddings and independence constraints.

Within MFD features, the biological features achieve the highest mean Area under the Receiver Operating Characteristic (AUROC) values in all but one setting (Table 1 and Supplementary Appendix Table A4). We observe that both technical and biological features achieve comparable results, pointing to the worrisome scenario where predictions of classifiers that do not explicitly account for sources of noise may be based on artifacts rather than biology. However, our disentangled biological features do surpass the technical ones, and combining both feature subspaces does not yield better performance than the biological features alone. This underlines the success of our DRL strategy and indicates that the biological features generalize better. Furthermore, compared to a “raw,” unregularized baseline model, MFD retains the predictive performance, while offering increased interpretability.

**Table 1.** Results of the enhancer classification task on the FANTOM5 dataset.

*λ*_indep_	Combined	Biological	Technical
Baseline	**0.68**		
0	0.66	0.67	0.66
0.001	0.67	**0.68**	0.62
0.01	0.63	0.64	0.61
0.1	0.58	0.58	0.56

For each available tissue type we train a range of logistic regression models using different features obtained from pretrained MFD models and report mean AUROC values computed across all tissue types. Bolded values indicate the best scores. We found that biological MFD features alone are as predictive as features from an unconstrained baseline model.

### 3.3 Biological features improve VEP

We further evaluate the utility of MFD in a zero-shot VEP task. Selecting the model pretrained with *λ*_indep_ of 0.001, based on its performance in the enhancer prediction task on the FANTOM5 dataset (Section 3.2), we encode for each variant its corresponding reference and alternative sequences, obtaining features $s_{\text{full}}^{\text{ref}}=[s_{\text{bio}}^{\text{ref}},s_{\text{tech}}^{\text{ref}}]$ and $s_{\text{full}}^{\text{alt}}=[s_{\text{bio}}^{\text{alt}},s_{\text{tech}}^{\text{alt}}]$. VEPs are then calculated as the difference in model predictions: $\Phi (s_{\text{full}}^{\text{ref}})-\Phi (s_{\text{full}}^{\text{alt}})$, where $\Phi_{i}(x)=\sigma (xw_{i}+b_{i})$ is the prediction for the *i*th output class (see Equation (1) and Fig. 1). We further obtain VEPs for the biological signal by calculating predictions for the alternative allele as $\Phi ([s_{\text{bio}}^{\text{alt}},s_{\text{tech}}^{\text{ref}}])$, i.e. using biological features for the alternative allele sequence and technical features for the reference one (and vice versa for the technical VEPs). We average model predictions across small shifts around the center and average the predictions for the forward and reverse strands.

**Figure 4.**
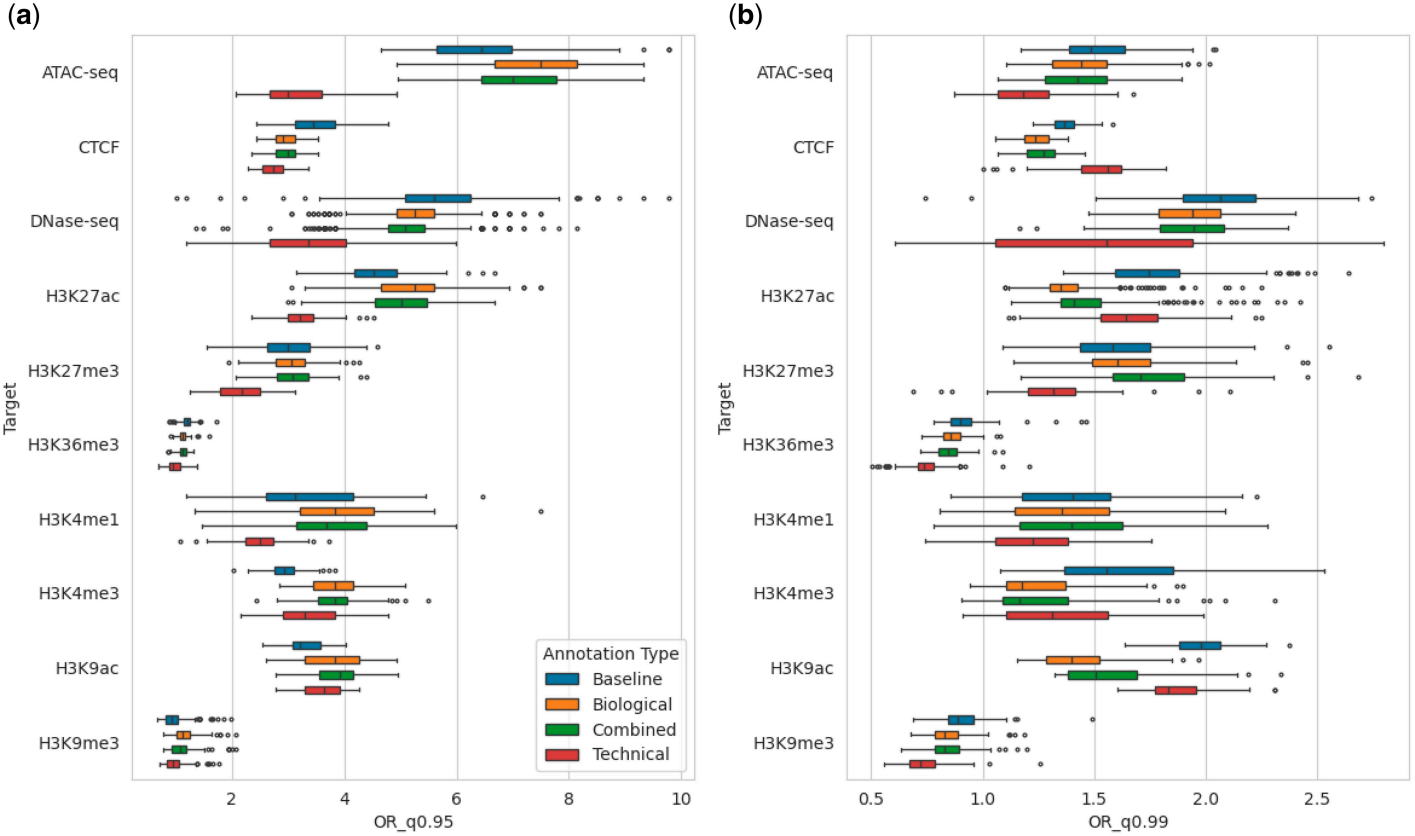
Mean odds ratios of identifying: (a) an eQTL variant in the GTEx dataset and (b) a rare variant in the gnomAD dataset, over different assays and targets for different feature types. We use the upper 95th and 99th quantiles as the cutoff for identifying the variants for GTEx and gnomAD, respectively.

By choosing a cutoff value based on the quantiles of the resulting distribution of VEPs, we perform zero-shot variant discoveries for Expression Quantitative Trait Loci (eQTL) variants in the Genotype-Tissue Expression (GTEx) (Lonsdale *et al.* 2013), and rare PLS-CRE variants in the gnomAD (Benegas *et al.* 2023) datasets, which we describe in more detail in Supplementary Appendix Sections F.1 and F.2. We compute the overall enrichment per VEP annotation type by aggregating the tagged variants across all 2106 outputs (Table 2). For the first two quantile cutoffs, all feature types yield comparable Odds Ratios (ORs); for the most extreme cutoffs, the technical annotations achieve a 7% lower enrichment for both datasets. Features from the baseline model yield no improvement over the combined or biological ones in all the settings. Overall, the biological annotations yield an improvement over the baseline in all quantile settings in both datasets. To gain insights into potential class biases, we plot the mean ORs using VEPs corresponding to predictions within each target assay in Fig. 4. The combined and biological VEPs consistently yield comparable enrichment values, while the technical ones vary more strongly across targets.

**Table 2.** Enrichments of: (a) an eQTL variant in the GTEx dataset and (b) a rare variant in the gnomAD dataset, over all experiment outputs per-feature (combined, biological, and technical predictions).

Quantile	Annotation	Enrichment
**(a)**		
0.9	Baseline	1.04
	Biological	1.05
	Combined	1.05
	Technical	1.05
0.95	Baseline	1.12
	Biological	1.13
	Combined	1.12
	Technical	1.14
0.99	Baseline	1.42
	Biological	1.43
	Combined	1.42
	Technical	1.32
**(b)**		
(0.1, 0.9)	Baseline	1.15
	Biological	1.16
	Combined	1.16
	Technical	1.16
(0.01, 0.99)	Baseline	1.27
	Biological	1.28
	Combined	1.29
	Technical	1.26
(0.001, 0.999)	Baseline	1.71
	Biological	1.77
	Combined	1.78
	Technical	1.66

The values are computed over the total numbers of unique true positive and false positive variants identified. MFD-derived features improve performance over the baseline, while allowing for greater interpretability—separating the biological and technical factors shows that albeit the technical features are predictive, the biological ones alone suffice for good performance.

## 4 Discussion

MFD is a deep learning model designed to learn a disentangled representation of the human epigenome, trained to isolate low-dimensional biological features from those of a technical nature. On several independent downstream tasks, we demonstrated that predictive models utilizing the biological features outperform those that incorporate technical features or a combination thereof. This finding substantiates the model’s capability to effectively separate technical biases inherent in the training data from genuine biological signals, thereby enhancing the accuracy of DNA sequence-based predictions through effective “de-noising.” The task of enhancer prediction presented a considerable challenge, primarily due to the complex and nuanced nature of gene regulation syntax. This complexity is reflected in the sub-optimal average AUROCs observed for enhancer classification tasks. Nevertheless, we demonstrated that MFD-derived biological features are sufficient to achieve the predictive performance of an unconstrained baseline model while offering greater interpretability. In the VEP task, features derived from diverse experiments demonstrated variable success in identifying true variants, underscoring the profound impact of technical biases on prediction outcomes. However, when quantifying the overall enrichment, the MFD biological features consistently yielded better performance than the baseline model. Despite the considerable predictive power of technical features in several cases, we argue in favor of utilizing disentangled biological representations. By investigating model attribution maps, we showed how biological features attend to meaningful information (e.g. TF motifs) in a DNA sequence, in contrast to the unspecific attributions for technical features.

## Supplementary Material

btae403_Supplementary_Data

## Data Availability

The data underlying this article are available in Github, at https://github.com/HealthML/MFD. The datasets were derived from sources in the public domain: Encyclopedia of DNA Elements (ENCODE), https://doi.org/10.1093/nar/gkz1062.
