# Supplementary material for: Metadata-guided feature disentanglement for functional genomics
Source: Bioinformatics. 2024 Sep 4;40(Suppl 2):ii4–ii10. doi: 10.1093/bioinformatics/btae403 (PMC11373386; doi:10.1093/bioinformatics/btae403)
Supplement: btae403_Supplementary_Data [file btae403_supplementary_data.pdf]

Table 3. Metadata features from ENCODE experiments used for training of Metadata-guided Feature Disentanglement (MFD) models.

Feature	Direct	Interact.	Example Values
Biosample Term	No	Yes	chorionic villus, right lung
Biosample Organ	No	Yes	intestine, spleen
Biosample Life Stage	No	Yes	adult, embryonic
Age	No	Yes	10, 55
Age Unit	No	Yes	week, year
Target	Yes	Yes	Acc. DNA, H3K27ac
Assay	No	Yes	Dnase-seq, ATAQ-seq
GC-mean	Yes	Yes	0.43, 0.57
Lab	No	Yes	Bing-Ren, Bernstein
Year Released	No	Yes	2013, 2016

A. Model Training

We train MFD models on dinucleotide sequences of length 1152 from the GRCh38 human reference genome data, to predict peak-calls in $2,106$ tissue-specific DNA-accessibility (ATAC-seq, DNase-seq) and chromatin modification (ChIP-seq) experiments on human samples from the ENCODE database. We left out data from the 9th and 10th chromosomes as test data, and take 5% of the remaining samples as validation data. We augment the training data by randomly sampling either the forward or reverse complement of sequences, and applying random shifts of up to 8bp in either direction [Kelley et al., 2018, Avsec et al., 2021a]. The models are optimized for 100 epochs using the AMSGrad variant of the Adam optimizer [Reddi et al., 2019, Kingma and Ba, 2014] with a mini-batch size of 4096 and a learning rate of 10^{-3}. We monitor the AUROC values of validation set predictions after each training epoch, and use the model weights with highest AUROC values for downstream tasks. We set $C = 128$ as the dimensionality of the latent subspaces of the biological and technical features, and train 3 model variants, with the regularization coefficients for the subspace independence penalty $\lambda_{indep} \in \{10^{-3}, 10^{-2}, 10^{-1}\}$. The metadata variables used for training the models are listed in Table 3. The backbone model for sequence feature extraction was based on the Basenji2 architecture [Avsec et al., 2021a] (Figure 5). Model definition and training were implemented using the PyTorch and Pytorch-Lightning frameworks [Paszke et al., 2019, Falcon and The PyTorch Lightning team, 2019].

B. Evaluating Subspace Independence

We compare the effect of enforcing independence between the subspaces with an adversarial predictor (Section 2.2), which can capture non-linear dependencies, to a linear constraint, which penalizes the Frobenius norm of the cross-covariance matrix between the two features sets:

$$\mathcal{L}_{lin.indep.} = \|\mathrm{cov}(\mathbf{s}^{(1)}, \mathbf{s}^{(2)})\|_F \quad (5)$$

To quantify independence, we employ a batch-shuffling approach Belghazi et al. [2018] by training a Random Forest classifier to distinguish between pairs of $(\mathbf{s}_i^{(1)}, \mathbf{s}_i^{(2)})$ and $(\mathbf{s}_i^{(1)}, \mathbf{s}_{shuff(i)}^{(2)})$, where $\mathbf{s}_{shuff(i)}^{(2)}$ contains elements of $\mathbf{s}^{(2)}$ with a randomly shuffled order of rows (observations). By shuffling the order, we simulate drawing samples from $\mathbf{s}^{(2)}$ independently of $\mathbf{s}^{(1)}$. If $\mathbf{s}^{(1)} \perp \mathbf{s}^{(2)}$, then we would have $P(\mathbf{s}_i^{(1)}, \mathbf{s}^{(2)}) = P(\mathbf{s}^{(1)})P(\mathbf{s}^{(2)})$. We compare the achieved Random Forest accuracies and the AUROC scores of the corresponding model predictions for a set of models trained with different λ_{indep} values and independence constraints (Figure 6). All models trained with the adversarial constraint achieved a RandomForest accuracy of 50%, meaning the classifier was not able to distinguish between the original and shuffled subspaces. Enforcing just a linear independence resulted in an increase of the score to 75%, whereas a model without any constraint ($\lambda_{indep} = 0$) had a score of almost 90%. For $\lambda_{indep} < 0.1$ the adversarial penalty achieved comparable AUROC performance to both the linear and unconstrained model. Instead, we found a larger drop in AUROC, as compared to the baseline model, stemming from employing the metadata embeddings themselves.

C. Querying and processing ENCODE data

We queried the ENCODE database on the 27th of May 2021 and identified peak-calls from tissue-based experiments in human (assmembly GRCh38) samples. We considered only DNase-seq, ATAC-seq, CTCF ChIP-seq, and ChIP-seq for histone modifications (H3K27ac, H3K27me3, H3K9me3, H3K36me3, H3K4me1, H3K4me2, H3K4me3). We selected (pseudo-)replicated peaks for ATAC seq, IDR-thresholded peaks for CTCF and (pseudo-)replicated peaks for histone modifications. For DNase-seq, we considered all available peak files for a given experiment accession (with reported FDR = 0.05) because ENCODE did not provide (pseudo-)replicated or IDR-thresholded peaks for these experiments. Peak files were downloaded in narrowPeak format.

We queried metadata (e.g., sample quality metrics or ontologies) for all experiments using the ENCODE REST API. For experiment we queried attributes of the linked Biosample, Library and Experiment objects. A Biosample relates to a unique sample of biological material. A Library is a unique sequencing library (a sample of processed DNA for sequencing), and an Experiment encompasses a group of one or more experimental replicates. From the Biosamples, we queried the life_stage, age, and age_units attributes. We further retrieved standardized ontological terms describing the tissue for each experiment, specifically the term_name (e.g., "heart right ventricle") and the organ_slims (e.g., "heart"). These attributes are hierarchical, i.e., multiple term_names may map to the same organ_slim, and a term name may have more than one organ_slim (e.g., "intestine,large intestine"). Additionally, we queried metadata related to sample quality (e.g., the reported fraction of reads in peaks (FRIP)), the lab that produced the data, and the date the experiment was released.

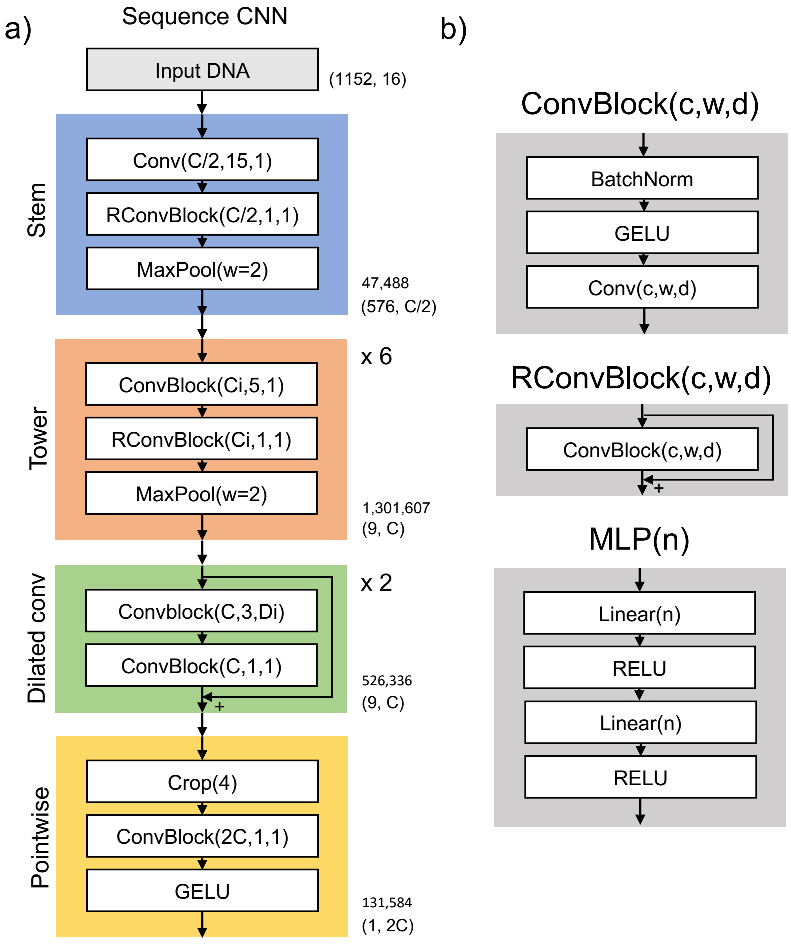

Fig. 5: **DNA-sequence convolutional neural network architecture and building blocks.** This figure has been adapted from ref Avsec et al. [2021a], where it was published under CC-BY 4.0 license (`https://creativecommons.org/licenses/by/4.0/`). Changes were made to reflect the parameters used and the new MLP building block. **a)** The DNA-sequence CNN is divided into four modules. The stem acts as a short motif-finder (C/2 channels). The tower grows the number of channels to C and reduces the spatial dimension/resolution. The dilated convolutions aggregate context across the sequence. The pointwise convolution transforms the sequence to its final intermediate representation with 2C channels. The numbers in brackets next to boxes denote the dimensions (the first is the spatial dimension, the second the number of channels). The number of parameters of each module is shown above the brackets. Panel **b)** shows the implementation of the building blocks of panel a). BatchNorm: Batch normalization, GELU: Gaussian Error Linear Unit, RELU: Rectified Linear Unit, MLP: Multilayer Perceptron, MaxPool(w): MaxPooling with stride and width w, Conv(c,w,d): convolution with c channels, kernel width w, and dilation d. Linear(n): Fully connected layer with n outputs. All experiments used C = 128.

We defined genomic regions of interest based on a set of peak files. This strategy was designed to be robust to within-sample outliers (extremely broad peaks) and between-sample outliers (extreme number of peaks). Processing is performed within groups (DNase, ATAC, separate histone modifications, CTCF). First, only peaks on autosomes and chromosome X are retained and peaks overlapping blacklisted regions are excluded (these include the ENCODE blacklist [Amemiya et al., 2019] and a small set of Vista enhancers [Visel et al., 2007b] used in downstream tasks). For each peak file, we calculated $w_{\max}$ as the 75th percentile plus 1.5 times the IQR of the peak width. If that value was shorter than 1000, it was set to 1000. Peaks that were longer than $w_{\max}$ were clipped so that their start and end coordinates did not extend any further than $w_{\max}$ away from the reported peak center (this caps the maximum peak length at $2w_{\max}$). For each peak file, we calculated $w_{\max}$ as the 75th percentile plus 1.5 times the IQR of peak width, with a minimum value of 1000. Peaks exceeding $w_{\max}$ were trimmed to a maximum length of $2w_{\max}$ from the peak center. Similarly, for each sample group, $p_{\max}$ was determined as the 75th percentile plus 1.5 times the IQR of peak counts. Files exceeding $p_{\max}$ peaks were reduced to only the strongest $p_{\max}$ peaks based on signal value.

For every sliding window within a set of regions of interest, I calculated overlaps to the original (i.e., non-filtered) peaks. Each peak file is considered its own class. If a region of interest is covered at least 50% by a peak from a specific file (i.e., experiment/replicate), it is considered a positive for that class (1),

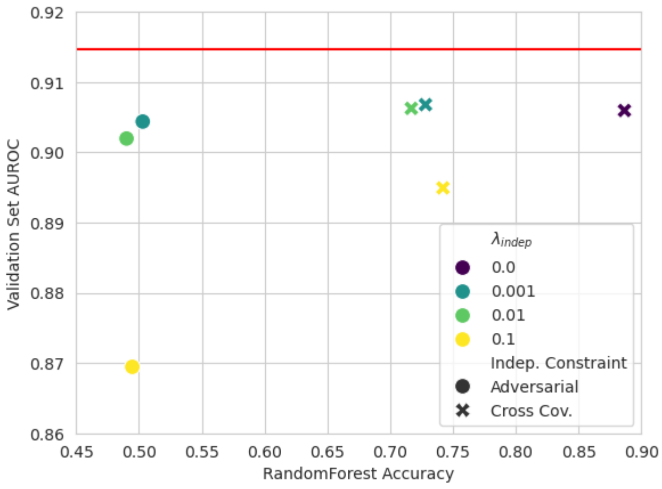

Fig. 6: Comparison of independence between latent subspaces (x-axis), measured by the accuracy of predicting between pairs of original and shuffled batches, and model predictive performance (y-axis) as AUROC of validation samples, for different regularization strengths λ_{indep} and independence constraints. The horizontal red line indicates the performance of a baseline model, without metadata embeddings and independence constraints.

otherwise it is considered a negative (0) [Zhou and Troyanskaya, 2015].

D. Interpretability

Figure 7 shows absolute (left column) and average (right column) contributions wrt. the center of the input sequence per target and assay type, for random negative sequences from the training data (first row), sequences centered around heart TF footprints (second row), and CTCF motifs (third row). While the features react differently to the different input sequences, they all exhibit asymmetrical behavior wrt. the sequence center, as well as periodicity in peaks. We hypothesize that these are artifacts caused by the architecture of the CNN model, since they are present both in contributions for meaningful sequences (heart TFs, CTCF), as well as in the negative sequences (with fewer than two corresponding experimental peaks in ENCODE). We thus treat them as a "baseline" signal which we subtract when interpreting the motif contributions in Figure 3. Further investigation of these artifacts - e.g., whether they persist regardless of the employed CNN backbone - is an interesting direction for future work.

E. Enhancer Prediction

We used the scikit-learn Python package [Pedregosa et al., 2011] to fit the logistic regression models. The models were optimized for a maximum of $1,000$ iterations per model, using balanced class weights. For each tissue type, we selected 80% of samples as training data and the remaining 20% for evaluation. We tuned the weights for the l_2 penalty of the logistic regression models with cross-validation on the training subset and evaluated the best-performing model on the test subset.

Table 4. Results of the enhancer classification task on the VISTA dataset. For each available tissue type, we train a range of logistic regression models using different features obtained from pretrained MFD models. We report mean AUROC values computed over all tissue types.

λ_{indep}	Combined	Biological	Technical
Baseline	**0.65**	-	-
0	**0.65**	**0.65**	0.64
0.001	0.64	**0.65**	0.62
0.01	0.64	0.64	0.62
0.1	0.55	0.55	0.57

E.1. FANTOM5

We used sequences from the 9th and 10th chromosomes of the FANTOM5 dataset [Dalby et al., 2017]. Enhancer sequences in this dataset were identified by an independent experimental assay of ENCODE, therefore it does not contain the exact same biases as the experiments in ENCODE. We further filtered the samples to match the experiments from ENCODE, containing at least 30 positive (enhancer) samples, and obtained the final set of 1459 enhancer sequences from 13 different tissues.

E.2. VISTA

We downloaded $1,940$ human sequences from the Vista Enhancer Browser [Visel et al., 2007a][1], which contains 998 enhancer sequences, and converted them to hg38 coordinates using the liftOver tool [Hinrichs et al., 2006]. These sequences were experimentally tested to have enhancer activity using a reporter assay and therefore, similar to the FANTOM dataset, VISTA enhancers are independent of biases present in ENCODE data. We selected tissue types with at least 50 positive (enhancer) samples. Since most sequences are longer than the input length of the MFD model, which has a median length of $1,530$ dinucleotides, we encoded sub-sequences from each sample using a sliding window approach and took the mean of the resulting features as inputs for the logistic regression models. We report the mean AUROC score computed over all tissue types in Table 4.

F. Variant Effect Prediction

F.1. GTEx

We retrieved fine-mapped GTEx Lonsdale et al. [2013] eQTL variants from the eQTL catalog Kerimov et al. [2023]. We constructed a positive set of likely causal fine-mapped variants, and a matched negative set, as follows: we excluded all variants that overlap protein-coding genes to limit variants acting through mechanisms other than transcription. For other types of transcripts (e.g., lncRNAs), we exclude variants that physically overlap the transcripts they are associated with. For the positive set, for every tissue, we keep only variants with a posterior inclusion probability (PIP) of > 0.95. If variants are also detected in other tissues, we keep them only if they have an average PIP of > 0.5 across all tissues. To sample the negative set, for each positive variant, we identify other variants within a $\pm$5kb window that do not overlap the gene the positive variant is associated with and had PIP < 0.05 in all tissues. We then select the variant with the most similar allele frequency to the positive variant. If

[1] We downloaded the data on 9th August 2021

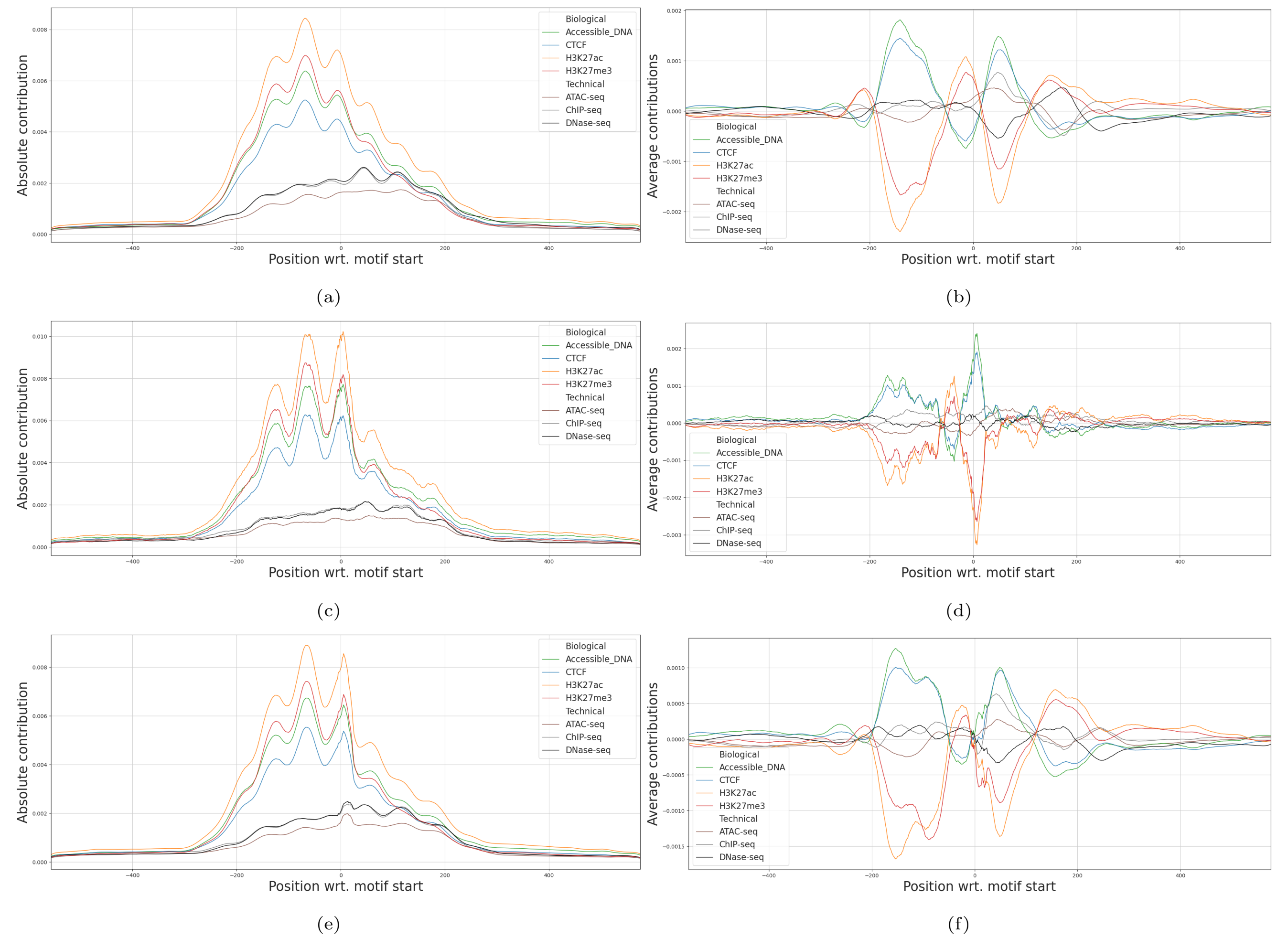

Fig. 7: **Contribution scores for direct features for targets and assays (a)** absolute and **(b)** average scores per base pair positions for 4,569 sequences of negative samples **(c)** absolute and **(d)** average scores per base pair positions for 100 sequences with TF footprints for heart tissue **(e)** absolute and **(f)** average scores per base pair positions for 4,457 sequences with CTCF motifs. In all cases we observe periodicity of peaks and asymmetry wrt. the center of the sequence, which we attribute to the workings of the underlying CNN model due to their prevalence across all input types.

there are ties based on the allele frequency, we choose the variant that is physically closest to the positive variant within the allele-frequency-matched variants. This selection results in a position- and allele-frequency-matched set of $2,304$ negative and $2,339$ positive single nucleotide variants (it can happen that the same negative variant is selected for multiple positive variants across tissues).

We perform variant effect prediction for all variants in this set and the $2,106$ model outputs. We also select the top 10%, 5% and 1% largest absolute variant effect predictions for each output, and calculate the enrichment (OR) of positive vs negative variants against all other variants. We use Fisher's exact test to determine significance.

F.2. gnomAD

We retrieved functionally annotated autosomal genetic variants from Hugging Face `https://huggingface.co/datasets/songlab/human_variants` as presented in Benegas et al. [2023]. These variants contain common variants (Minor Allele Frequency (MAF) $> 5\%$) as well as a matched number of rare singleton variants from gnomAD Chen et al. [2023]. We intersect these variants with ENCODE promoter-like cis-regulatory elements Moore et al. [2020]. $44,062$ variants remain after intersection, of which $26,112$ are rare and $17,950$ are common.

We predict variant effects for all variants and $2,106$ model outputs. For every model output, we calculate variant effect prediction cutoffs at varying thresholds (e.g., the top 0.1% and 0.01% most negative/positive values), and calculate odds ratios to quantify the enrichment for rare variants in those extremes vs all other remaining variants. We perform Fisher's exact tests to identify significant differences.
